# Pre-medication with oral anticoagulants is associated with better outcomes in a large multinational COVID-19 cohort with cardiovascular comorbidities

**DOI:** 10.1007/s00392-021-01939-3

**Published:** 2021-09-21

**Authors:** Marina Rieder, Nadine Gauchel, Klaus Kaier, Carolin Jakob, Stefan Borgmann, Annika Y. Classen, Jochen Schneider, Lukas Eberwein, Martin Lablans, Maria Rüthrich, Sebastian Dolff, Kai Wille, Martina Haselberger, Hanno Heuzeroth, Christoph Bode, Constantin von zur Mühlen, Siegbert Rieg, Daniel Duerschmied

**Affiliations:** 1 Department of Medicine III (Interdisciplinary Medical Intensive Care), Medical Center, Faculty of Medicine, University of Freiburg, Freiburg, Germany; 2 Department of Cardiology and Angiology I, Heart Center, University of Freiburg, Hugstetter Strasse 55, 79106 Freiburg, Germany; 3 Division of Infectious Diseases, Department of Medicine II, Medical Center, Faculty of Medicine, University of Freiburg, Freiburg, Germany; 4 Institute of Medical Biometry and Statistics, Faculty of Medicine, Medical Center-University of Freiburg, Freiburg, Germany; 5 Department I for Internal Medicine, Faculty of Medicine, University of Cologne, University Hospital Cologne, Cologne, Germany; 6 German Centre for Infection Research (DZIF), Partner Site Bonn-Cologne, Cologne, Germany; 7 Department of Infectious Diseases and Infection Control, Ingolstadt Hospital, Ingolstadt, Germany; 8 School of Medicine, Technical University of Munich, University Hospital Rechts der Isar, Munich, Germany; 9 4Th Department of Internal Medicine, Klinikum Leverkusen, Leverkusen, Germany; 10 Federated Information Systems, German Cancer Research Center, Heidelberg, Germany; 11 University Medical Center Mannheim, Mannheim, Germany; 12 Department for Internal Medicine II, Hematology and Medical Oncology, University Hospital Jena, Jena, Germany; 13 Leibniz Institute for Natural Product Research and Infection Biology, Hans-Knöll Institute, Jena, Germany; 14 Department of Infectious Diseases, West German Centre of Infectious Diseases, University Hospital Essen, University Duisburg-Essen, Duisburg, Germany; 15 University Clinic for Haematology, Oncology, Haemostaseology and Palliative Care, Johannes Wesling Medical Center Minden, University of Bochum, Bochum, Germany; 16 Department of Medicine I, Passau Municipal Hospital, Passau, Germany; 17 Department of Emergency and Intensive Care Medicine, Klinikum Ernst-Von-Bergmann, Potsdam, Germany

**Keywords:** COVID-19, Oral anticoagulation, SARS-CoV-2, Thrombosis

## Abstract

**Aims:**

Coagulopathy and venous thromboembolism are common findings in coronavirus disease 2019 (COVID-19) and are associated with poor outcome. Timely initiation of anticoagulation after hospital admission was shown to be beneficial. In this study we aim to examine the association of pre-existing oral anticoagulation (OAC) with outcome among a cohort of SARS-CoV-2 infected patients.

**Methods and results:**

We analysed the data from the large multi-national Lean European Open Survey on SARS-CoV-2 infected patients (LEOSS) from March to August 2020. Patients with SARS-CoV-2 infection were eligible for inclusion. We retrospectively analysed the association of pre-existing OAC with all-cause mortality. Secondary outcome measures included COVID-19-related mortality, recovery and composite endpoints combining death and/or thrombotic event and death and/or bleeding event. We restricted bleeding events to intracerebral bleeding in this analysis to ensure clinical relevance and to limit reporting errors. A total of 1 433 SARS-CoV-2 infected patients were analysed, while 334 patients (23.3%) had an existing premedication with OAC and 1 099 patients (79.7%) had no OAC. After risk adjustment for comorbidities, pre-existing OAC showed a protective influence on the endpoint death (OR 0.62, *P* = 0.013) as well as the secondary endpoints COVID-19-related death (OR 0.64, *P* = 0.023) and non-recovery (OR 0.66, *P* = 0.014). The combined endpoint death or thrombotic event tended to be less frequent in patients on OAC (OR 0.71, *P* = 0.056).

**Conclusions:**

Pre-existing OAC is protective in COVID-19, irrespective of anticoagulation regime during hospital stay and independent of the stage and course of disease.

**Graphic abstract:**

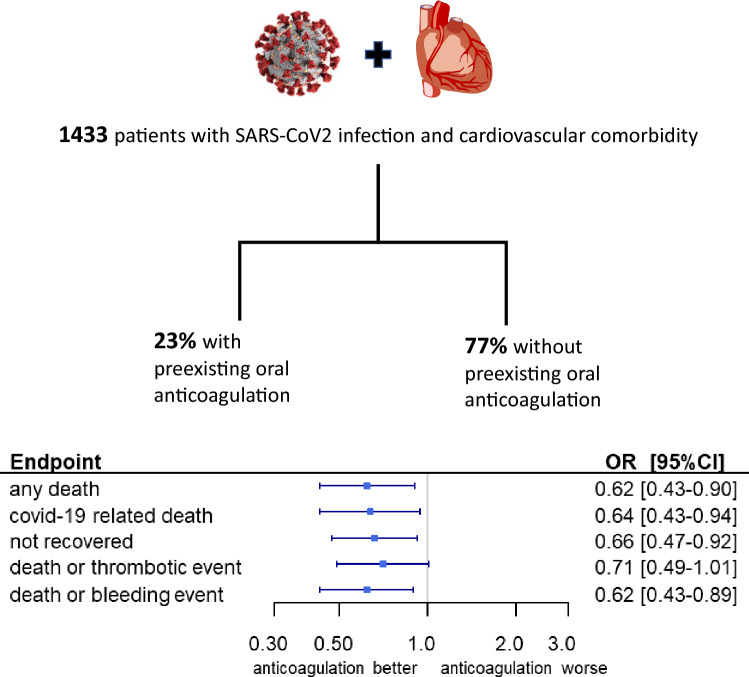

## Introduction

The novel coronavirus disease 2019 (COVID-19) is currently spreading rapidly, causing significant morbidity and mortality worldwide. An infection with the underlying severe acute respiratory syndrome coronavirus 2 (SARS-CoV-2) predominantly causes respiratory symptoms of varying degrees, ranging from mild dyspnoea to acute respiratory distress syndrome [1–3]. In addition, coagulopathy is a common and potentially outcome-limiting complication in SARS-CoV-2-infected patients, especially in severe cases [4, 5]. It has been argued that COVID-19 coagulopathy may differ from other causes of coagulopathy leading to venous thromboembolism (VTE), including important contribution of systemic inflammatory activation and endotheliitis, which may not be adequately targetable by conventional anticoagulation [6–8]. Consistent with that hypothesis, VTE has been observed in 30–60% of severe COVID-19 cases [9, 10], sometimes even despite therapeutic or prophylactic anticoagulation [10, 11]. Emerging evidence indicates that anticoagulation is nevertheless beneficial for patients with COVID-19, especially in severe cases [12–14]. The current consensus statements recommend a repeated risk assessment for VTE and bleeding, to enable timely diagnosis and adequate treatment of coagulation abnormalities in COVID-19 [15].

All these data and recommendations focus on anticoagulation during hospitalization; however, the association of pre-existing oral anticoagulation (OAC) with outcome parameters in COVID-19 is still unclear. Analysing relevant outcome parameters of patients pre-treated with OAC may provide essential information, because mainly older patients suffering from comorbidities such as chronic cardiovascular disease are at risk for fatal outcomes, a patient group also frequently pre-treated with OAC. Using the Lean European Open Survey on SARS-CoV-2 Infected Patients (LEOSS) registry [16], an extensive database on the clinical course of SARS-CoV-2 infected patients, we performed a retrospective risk-adjusted analysis to evaluate the association between pre-existing OAC and outcome in COVID-19.

## Patients and methods

### Study population

We here report data from the Lean European Open Survey on SARS-CoV-2 Infected Patients (LEOSS). LEOSS is a multi-centre, non-interventional registry study for the documentation of SARS-CoV-2 infected patients mandated by the Emerging Infections Task Force (EITaF) of the European Society for Clinical Microbiology and Infectious Diseases (ESCMID) and supported by the German Center for Infection Research (DZIF) and the German Infectious Disease Society (DGI).

Since LEOSS was initiated in March 2020, more than 120 sites predominantly located in Germany, but also in Austria, Belgium, Bosnia, Canada, Ireland, Italy, Latvia, Spain, Switzerland, Turkey, United Kingdom and the USA have retrospectively reported data on hospitalized and outpatient SARS-CoV-2 infected patients at all different stages of disease severity (from asymptomatic to life threatening). LEOSS is registered at the German Clinical Trials Register (DRSK, S00021145) and was approved by the applicable local ethics committees of all participating centers.

**Fig. 1:**
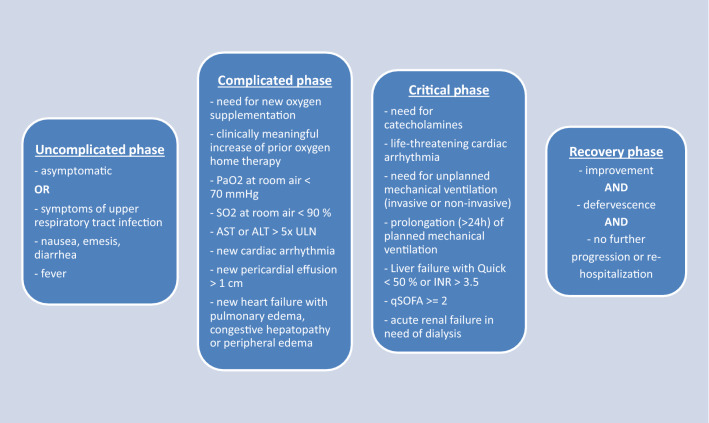
Definition of the different phases (uncomplicated, complicated and critical) of COVID-19 disease used for this analysis

The primary outcome was all-cause mortality. Secondary outcome measures were COVID-19-related mortality, recovery (defined as significant improvement of clinical status as defined in the design of the LEOSS registry, Fig. 1) and the composite endpoints death and/or thrombotic event and death and/or bleeding event. Bleeding event was characterized as intracerebral bleeding in this analysis. We restricted bleeding events to intracerebral bleeding in this analysis to ensure clinical relevance and to limit reporting errors. Other bleeding events were not systematically recorded in the LEOSS registry. The composite endpoints were chosen to inform about clinically relevant events and to account for expected low numbers of thrombotic or bleeding events in the examined cohorts. OAC was defined as premedication with any Vitamin-K antagonist or Non-Vitamin-K antagonist (rivaroxaban, apixaban, edoxaban, dabigatran etexilate). Indication for OAC premedication was not assessed systematically, but atrial fibrillation with increased risk for systemic embolization was likely a main reason for patients to take OAC.

### Data collection

The data were recorded at each site in an electronic case report form (eCRF) operated using the cohort platform ClinicalSurveys.net and software developed by Questback (Oslo, Norway). The data entry was conducted in an anonymized manner. Moreover, the LEOSS study team performed further data anonymization and categorization of data to prevent the possibility to draw conclusions to a single hospital.

### Data analysis

Difference between categorical and continuous variables were analysed using Fisher's exact test and Students’ *t* tests, respectively. Since our analysis was solely retrospective and patients were not randomized to the two treatment options (anticoagulation vs. no anticoagulation), multivariable logistic regression analyses were applied to verify the impact of anticoagulation. As potential confounders, a total of 10 baseline patient characteristics (all covariates listed in Table 1) were used. In detail, we considered age, gender, BMI and smoking status as well as the phase of disease at diagnosis and a number of pre-existing cardiovascular diseases, e.g. atrial fibrillation or coronary artery disease. Missing values for age (*n* = 6) were imputed using median imputation (median age = 70 years). For BMI and smoking status, missing values were more frequent and used as separate variables. For other pre-existing diseases, there were no codes to indicate that data were missing; thus if the patient’s electronic health record did not include information on a clinical characteristic, it was assumed that that characteristic was not present. No adjustment for multiple testing was carried out. Thus, p values may not be interpreted as confirmatory but are descriptive in nature and inferences drawn from the 95% confidence intervals may not be reproducible. All analyses were carried out using Stata 16.1 (StataCorp, College Station, TX, USA).

**Table 1:** Patient characteristics

	Without OAC (*n* = 1099)	With OAC (*n* = 334)	*P* value
Patients characteristics			
Age [years, mean ± SD]	69.56 ± 13.61	77.04 ± 10.31	** < 0.001**
Sex [male]	675 (61.42%)	188 (56.29%)	0,097
BMI [kg/m^2^]			
< 18.5	15 (1.36%)	3 (0.9%)	0.779
18.5–24.9	199 (18.11%)	83 (24.85%)	**0.008**
25–29.9	250 (22.75%)	81 (24.25%)	0.604
30–34.9	143 (13.01%)	38 (11.38%)	0.454
> 34.9	89 (8.1%)	11 (3.29%)	**0.002**
Unknown	403 (36.67%)	118 (35.33%)	0.697
Smoking status			
Smoker	80 (7.28%)	17 (5.09%)	0.173
Ex-smoker	115 (10.46%)	33 (9.88%)	0.837
Non-smoker	363 (33.03%)	105 (31.44%)	0.641
Unknown	541 (49.23%)	179 (53.59%)	0.170
Phase of disease at diagnosis			
Complicated	361 (32.85%)	114 (34.13%)	0.691
Critical	97 (8.83%)	24 (7.19%)	0.371
Medical history			
Solid tumour	137 (12.47%)	57 (17.07%)	**0.036**
Cardiovascular diseases			
Atrial fibrillation	142 (12.92%)	222 (66.47%)	** < 0.001**
Coronary artery disease	220 (20.02%)	123 (36.83%)	** < 0.001**
Prior myocardial infarction	94 (8.55%)	56 (16.77%)	** < 0.001**
Peripheral artery disease	67 (6.1%)	47 (14.07%)	** < 0.001**
Arterial hypertension	963 (87.63%)	277 (82.93%)	**0.035**
Cerebrovascular disease	137 (12.47%)	72 (21.56%)	** < 0.001**
Diabetes mellitus			
Without organ damage	229 (20.84%)	51 (15.27%)	**0.027**
With organ damage	116 (10.56%)	52 (15.57%)	**0.015**

Bold *P* values are statistically significant

*P* values refer to the comparison between the OAC and the non-OAC patients. The data are presented as mean ± standard deviation or on number of patients (with percentage based on the number of patients with a non-missing value for that characteristic). Comparisons are based on Student’s *t* test or on chi-square test/Fisher’s exact test as appropriate

## Results

### Patients

**Fig. 2:**
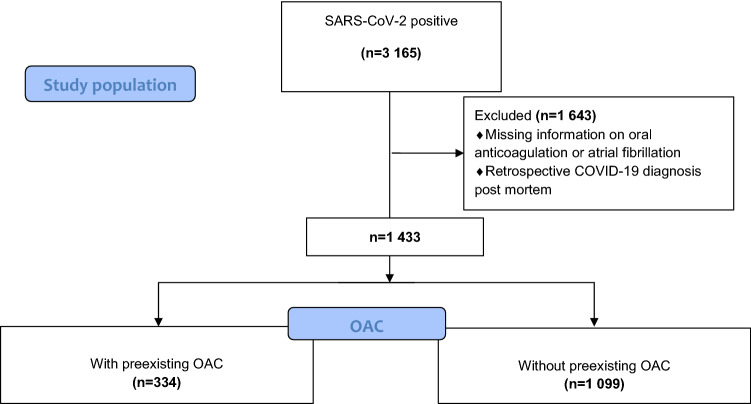
Schematic representation of the allocation to the cohort with or without pre-existing oral anticoagulation. Of the 3165 patients initially included in this analysis, we had to exclude 1643 patients due to missing information on oral anticoagulation or atrial fibrillation as a relevant comorbidity. Furthermore, all patients with retrospective COVID-19 diagnosis post mortem were excluded. Finally, 334 patients with pre-existing OAC and 1099 patients without pre-existing oral anticoagulation were included in the analysis. The flow diagram is based on the STROBE criteria for reporting of observational studies [38, 39]

The final anonymized dataset included 3 165 patients infected with SARS-CoV-2 at different phases of disease (uncomplicated, complicated, critical, recovery; Fig. 1). We had to exclude 1 643 patients because of missing information about pre-existing oral anticoagulation or atrial fibrillation or because of retrospective COVID-19 diagnosis post mortem. We included 1 433 patients in our analysis (Fig. 2). A total of 334 patients (23.3%) had an existing premedication with OAC, 1 099 patients (79.7%) had no OAC. Patients with pre-existing OAC were significantly older (77.0 years vs. 69.6 years, *P* < 0.001), tended to be more likely female (56.3% male with OAC vs. 61.4% male without OAC, *P* = 0.097) and had more comorbidities. They suffered significantly more frequently from solid tumours (17.1% vs. 12.5%, *P* = 0.036), coronary artery disease (36.8% vs. 20.0%, *P* < 0.001), prior myocardial infarction (16.8% vs. 8.6%, *P* < 0.001), atrial fibrillation (66.5% vs. 12.9%, *P* < 0.001), peripheral vascular disease (14.1% vs. 6.1%, *P* < 0.001) and cerebrovascular disease (21.6% vs. 12.5%, *P* < 0.001). Arterial hypertension was significantly more frequent in patients without OAC (87.6% vs. 82.9%, *P* = 0.035). The severity of disease at diagnosis was comparable in both groups, indicating similar grades of illness (complicated phase 34.1% in patients with OAC vs. 32.9% in patients without OAC, *P* = 0.691; critical phase 7.2% in patients with OAC vs. 8.8% in patients without OAC, *P* = 0.371; Table 1).

There were relatively large frequencies of unknown data on BMI (35.3% in the group with OAC and 36.7% in the group without OAC) and smoking status (53.6% in the group with OAC and 49.2% in the group without OAC).

### Unadjusted outcomes

Unadjusted outcomes did not differ significantly between patients with or without OAC. The rate of all-cause death and COVID-19 related death was slightly higher in patients with OAC without statistical significance (any death: 26.1% vs. 23.7%, *P* = 0.382; COVID-19 related death: 22.8% vs. 20.7%, *P* = 0.444). A similar proportion of patients recovered from COVID-19 in both groups (67.1% with OAC vs. 68.9% without OAC, *P* = 0.545). Thrombotic events occurred only in a few cases in both groups, with 4.2% in patients with OAC and 3.7% in patients without OAC, respectively (*P* = 0.745). Intracerebral bleedings occurred in 1.2% of patient without OAC and in 0.9% of patients with OAC in similar proportions (*P* = 1.0). Death or thrombotic event as combined endpoint occurred numerically slightly more often without statistical significance in the group with OAC (28.7% vs. 26.2%, *P* = 0.360) as well as the combined endpoint death or intracerebral bleeding (26.1% vs. 24.4%, *P* = 0.563; Table 2).

**Table 2:** Unadjusted outcomes

	Without OAC (*n* = 1099)	With OAC (*n* = 334)	*P* value
All-cause mortality	260 (23.66%)	87 (26.05%)	0.382
COVID-19 related mortality	227 (20.66%)	76 (22.75%)	0.444
Recovered	757 (68.88%)	224 (67.07%)	0.545
Thrombotic events	41 (3.73%)	14 (4.19%)	0.745
Intracerebral bleeding	13 (1.18%)	3 (0.90%)	1.000
Death or thrombotic event	288 (26.21%)	96 (28.74%)	0.360
Death or intracerebral bleeding	268 (24.39%)	87 (26.05%)	0.563

*P* values refer to the comparison between the OAC and the non-OAC patients. The data are presented as mean ± standard deviation or on number of patients (with percentage based on the number of patients with a non-missing value for that characteristic). Comparisons are based on Student’s *t* test or on chi-square test/Fisher’s exact test as appropriate

### Risk adjusted outcomes

**Fig. 3:**
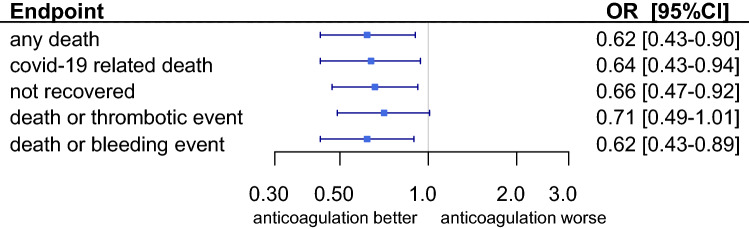
Risk-adjusted outcomes in patients pre-treated with oral anticoagulation and in a cohort without oral anticoagulation pre-treatment. The results of multivariate logistic regression analyses with 23 predefined baseline patient characteristics included as potential confounders (all covariates listed in Table 3)

After risk adjustment for comorbidities, a pre-existing OAC was associated with a significant better outcome regarding the endpoint all-cause death (OR 0.62, *P* = 0.013) as well on the secondary endpoint COVID-19 related death (OR 0.64, *P* = 0.023), non-recovery (OR 0.66, *P* = 0.014) and the combined endpoint death or intracerebral bleeding (OR 0.62, *P* = 0.01). Patients with pre-existing OAC tended to have a better outcome at the combined endpoint death or thrombotic event (OR 0.71, *P* = 0.056; Table 3, Fig. 3).

**Table 3:** Results of regression adjustment approach (*N* = 1433, logistic regression)

	Any death				Covid-19 death				Not recovered				Death or thrombotic event				Death or bleeding event			
	OR	*P* value	95% CI		OR	*P* value	95% CI		OR	*P* value	95% CI		OR	*P* value	95% CI		OR	*P* value	95% CI	
Oral anticoagulation	**0.62**	**0.013**	**0.43**	**0.90**	**0.64**	**0.023**	**0.43**	**0.94**	**0.66**	**0.014**	**0.47**	**0.92**	**0.71**	**0.056**	**0.49**	**1.01**	**0.62**	**0.010**	**0.43**	**0.89**
Age	1.08	** < 0.001**	1.06	1.09	1.08	** < 0.001**	1.06	1.09	1.05	** < 0.001**	1.04	1.06	1.06	** < 0.001**	1.04	1.07	1.07	** < 0.001**	1.06	1.09
Male	1.80	** < 0.001**	1.34	2.43	2.02	** < 0.001**	1.48	2.78	1.61	** < 0.001**	1.24	2.10	1.76	** < 0.001**	1.32	2.34	1.86	** < 0.001**	1.38	2.51
BMI < 18.5	0.88	0.841	0.26	3.04	0.74	0.663	0.19	2.90	0.88	0.821	0.28	2.71	0.77	0.669	0.23	2.59	0.85	0.801	0.25	2.95
BMI 18.5–24.9	0.82	0.344	0.54	1.24	0.70	0.115	0.45	1.09	0.96	0.824	0.66	1.40	0.83	0.347	0.55	1.23	0.85	0.457	0.57	1.29
BMI 25–29.9	1 (omitted)				1 (omitted)				1 (omitted)				1 (omitted)				1 (omitted)			
BMI 30–34.9	1.14	0.613	0.69	1.88	1.11	0.707	0.65	1.88	1.08	0.745	0.69	1.68	1.06	0.796	0.66	1.71	1.14	0.603	0.70	1.87
BMI > 34.9	1.35	0.349	0.72	2.51	1.64	0.125	0.87	3.10	1.24	0.435	0.72	2.15	1.46	0.195	0.82	2.59	1.26	0.460	0.68	2.34
BMI unknown	1.09	0.643	0.76	1.56	1.19	0.363	0.82	1.72	1.19	0.291	0.86	1.65	1.02	0.930	0.72	1.43	1.07	0.704	0.75	1.53
Smoker	1.08	0.805	0.60	1.93	0.90	0.742	0.48	1.68	0.94	0.810	0.56	1.58	0.92	0.763	0.52	1.61	0.97	0.931	0.55	1.74
Non-smoker	0.90	0.499	0.66	1.23	0.81	0.200	0.58	1.12	0.94	0.670	0.71	1.24	0.93	0.649	0.69	1.26	0.83	0.249	0.61	1.14
Ex-smoker	1.23	0.390	0.77	1.96	1.08	0.770	0.66	1.75	0.85	0.472	0.55	1.32	1.17	0.493	0.75	1.83	1.12	0.637	0.70	1.78
Smoking unknown	1 (omitted)				1 (omitted)				1 (omitted)				1 (omitted)				1 (omitted)			
Complicated phase at diagnosis	2.95	** < 0.001**	2.21	3.96	3.02	** < 0.001**	2.22	4.10	2.60	** < 0.001**	2.01	3.37	3.10	** < 0.001**	2.35	4.09	3.09	** < 0.001**	2.31	4.12
Critical phase at diagnosis	9.51	** < 0.001**	6.01	15.03	8.22	** < 0.001**	5.18	13.06	7.02	** < 0.001**	4.56	10.80	9.62	** < 0.001**	6.17	14.99	8.97	** < 0.001**	5.70	14.12
Solid tumour	1.32	0.149	0.91	1.92	1.31	0.178	0.89	1.92	1.21	0.277	0.86	1.71	1.16	0.428	0.80	1.67	1.27	0.211	0.87	1.84
Atrial fibrillation	1.54	**0.015**	1.09	2.18	1.47	**0.036**	1.03	2.11	1.56	**0.007**	1.13	2.16	1.39	0.056	0.99	1.95	1.48	**0.028**	1.04	2.09
Coronary artery disease	1.16	0.394	0.83	1.63	1.23	0.241	0.87	1.75	1.09	0.572	0.80	1.50	1.15	0.419	0.82	1.59	1.15	0.410	0.82	1.61
Prior myocardial infarction	1.03	0.900	0.65	1.63	1.04	0.866	0.65	1.68	1.11	0.633	0.72	1.70	0.96	0.875	0.62	1.51	1.01	0.970	0.64	1.60
Peripheral artery disease	0.77	0.301	0.47	1.26	0.80	0.379	0.48	1.32	0.73	0.169	0.46	1.15	0.92	0.722	0.58	1.46	0.76	0.271	0.47	1.24
Arterial hypertension	0.87	0.513	0.58	1.31	0.91	0.654	0.59	1.39	0.97	0.882	0.67	1.40	0.91	0.645	0.62	1.35	0.87	0.511	0.59	1.31
Cerebrovascular disease	0.99	0.941	0.68	1.42	0.97	0.859	0.66	1.41	1.06	0.755	0.75	1.48	0.98	0.923	0.69	1.40	0.96	0.833	0.67	1.38
Diabetes without organ damage	1.24	0.231	0.87	1.77	1.28	0.190	0.89	1.85	1.19	0.271	0.87	1.64	1.15	0.429	0.82	1.61	1.24	0.237	0.87	1.75
Diabetes with organ damage	1.98	** < 0.001**	1.35	2.90	1.92	**0.001**	1.30	2.85	1.71	**0.004**	1.19	2.45	1.84	**0.001**	1.27	2.67	1.90	**0.001**	1.30	2.79

Bold *P* values are statistically significant

Analysing the different comorbidities and its risk-adjusted influences on the different endpoints, the complicated or critical stage of disease had a significant influence on all endpoints for a worse outcome. Patients with atrial fibrillation had a significant higher risk for mortality (OR 1.54, *P* = 0.015), whereas patients with coronary artery disease (OR 1.16, *P* = 0.394) or prior myocardial infarction did not (OR 1.03, *P* = 0.9). Also patients with peripheral vascular disease (OR 0.77, *P* = 0.301), cerebrovascular disease (OR 0.99, *P* = 0.941) or hypertension (OR 0.87, *P* = 0.513) did not have an increased risk of mortality. If patients had diabetes mellitus with organ damage, risk for mortality was significantly increased (OR 1.98, *P* < 0.001), whereas patients with diabetes mellitus without organ damage did not (OR 1.24, *P* = 0.231). Age and sex (being male has a higher mortality, OR 1.8, *P* < 0.001; Table 3) had a significant worsening influence on all endpoints. In our analyses we did not see a significant influence of the smoking status or the BMI of the patients on all endpoints. For further information on the influence of the different covariates see Table 3.

## Discussion

In this retrospective analysis of the LEOSS registry, an anonymized multi-center registry for the documentation of SARS-CoV-2 infected patients, we studied outcome with regard of pre-existing oral anticoagulation (OAC) irrespective of anticoagulation during hospital stay. In this real-world cohort, pre-existing OAC was associated with less mortality and increased recovery rate.

The hypothesis that pre-existing OAC might have an effect on the outcome of COVID-19 was based on the recent findings on thrombotic events and anticoagulation during SARS-CoV-2 infection [17–19]. Thromboembolic complications have emerged as a common and often limiting complication in COVID-19. Since first case series from Wuhan reported an association between pulmonary embolism and COVID-19 [20], various studies described that patients suffering from COVID-19 present a high rate of venous and arterial thromboembolic events, partly even despite anticoagulation at prophylactic or therapeutic doses [10, 21, 22]. It has been suggested that hyperinflammation and hypoxemia lead to endothelial dysfunction and endotheliitis and as a consequence to enhanced risk for thrombus formation [23–27]. The increased incidence of VTEs in patients with COVID-19 despite anticoagulation and the occurrence of major bleeding in up to 5.6% of severe cases has stimulated a debate about the ideal anticoagulation scheme [28]. Furthermore, a recent trial showed an increase in oral anticoagulant plasma levels in patients with SARS-CoV-2 infection treated with antiviral agents [29]. However, recent studies indicated that the benefits of anticoagulation during hospital-stay outweighed the risk of bleeding: Tang et al. reported that patients with severe COVID-19 receiving anticoagulation with unfractionated or (in most cases) low-molecular weight heparin appeared to have a lower mortality than patients without heparin therapy [13]. Nadkarni et al. described that anticoagulation during hospital stay was associated with lower mortality and intubation in COVID-19 and therapeutic anticoagulation tended to be more efficient than prophylactic doses [14]. Yet, most available data on the effects of anticoagulation in COVID-19 are based on the anticoagulation during hospital stay.

Few recent studies showed inconclusive and oppositional results of mortality in patients with SARS-CoV-2 infection on pre-existing oral anticoagulation. One study from Spain showed worse outcome in patients on oral anticoagulation with a surprisingly high mortality rate of 68.2% in this cohort[30]. In contrast two studies from Italy showed improved outcomes of patients on pre-existing oral anticoagulation [17, 31]. Other studies from Italy and Sweden did not show any protective or worsening effect of oral anticoagulation treatment [32, 33].

Our colleagues could show elevated markers of thrombo-inflammatory activation in patients with cardiovascular diseases and their prediction for a worse outcome in the population of the LEOSS registry [34], reinforcing the benefit of early or even pre-existing anticoagulation in these patients in line with our findings in the same population.

We aimed to evaluate the effects of pre-existing OAC on prognosis and outcome of COVID-19 of all stages of disease irrespective of anticoagulation regime during inpatient treatment and irrespective of the stage and course of SARS-CoV-2 infection. Patients pre-treated with OAC are more likely to suffer from cardiovascular comorbidities that make them susceptible for a more severe course of COVID-19 [35]. The findings of our analysis are in line with this hypothesis, showing that patients with OAC were older and had higher rates of atrial fibrillation and other cardiovascular comorbidities. Despite this, mortality rates did not differ between both cohorts in the unadjusted outcome analysis. After risk adjustment, a protective effect of a pre-existing OAC became evident: pre-existing OAC was associated with significantly reduced all-cause and COVID-19-related mortality and improved the recovery rate. Atrial fibrillation itself shows in our covariate adjustment a negative influence on all outcomes. As shown in Table 1, a substantial part of the patients hospitalized with OAC was not diagnosed with atrial fibrillation. At the same time, a substantial part of the patients that was diagnosed with atrial fibrillation was not treated with OAC at hospital admission. In a real-world cohort like ours, this seems realistic as patients with atrial fibrillation might have discontinued medication and patients undergoing OAC treatment might have had other underlying diseases than atrial fibrillation (e.g. thrombosis, mechanical heart valves). As a result, we believe it is of special interest to identify the independent contribution of each predictor: “What is the impact of OAC treatment independent of the patients sex, age, atrial fibrillation and stage of disease?” and “What is the impact of atrial fibrillation independent of OAC treatment, sex, age and stage of disease?”. To answer both questions in one regression model, we believe our methodology is most appropriate compared to other statistical methods [36]. Notably our results were independent of in-hospital anticoagulation regime and irrespective of the clinical stage and course of COVID-19 disease. Taken together, we show with the data from a large multi-national cohort study that a pre-existing oral anticoagulation has a protective effect on the outcome of COVID-19.

## Limitations

Apart from the limitations commonly associated with retrospective studies, the LEOSS registry has some specific limitations. Despite its multinational approach, most patients were documented in Germany and a generalization of our results could be biased. Furthermore, the data on the extent of underlying comorbidities or regarding substance, dose or duration of treatment with OAC were not incorporated in the LEOSS database, as both, comorbidities and medication intake, were collected as binary categories. Numerous patients had to be excluded due to missing data on anticoagulation.

Furthermore the anticoagulation regime during hospitalization was not recorded systemically. The lack of knowledge how and if patients were anticoagulated during the hospital stay significantly limits the interpretation of the data, e.g. whether anticoagulation was reduced in prior anticoagulated patients or whether previously non-anticoagulated patients received "full" anticoagulation as a prevention measure. Anonymization and categorization of data made it impossible to consider parameters such as length of hospital stay or length of mechanical ventilation as outcome parameters. Furthermore, major bleeding events such as gastrointestinal bleedings have not been recorded systematically in the LEOSS registry, only intracerebral bleedings were considered systematically in the database. Furthermore the chosen endpoint "intracranial bleeding" occurred only 3 times the anticoagulated group, a number way too small to draw valuable conclusions.

## Conclusion

In this observational study evaluating real-world data on the pre-existing OAC on outcome in COVID-19, we investigated mortality, recovery, thrombotic events and intracerebral bleeding using data from the Lean European Open Survey on SARS-CoV-2 Infected Patients (LEOSS). After risk adjustment, a substantial decrease in all-cause and COVID-19-related mortality and an increased recovery rate in patients with pre-existing OAC compared to patients without pre-existing OAC was observed. The combined outcome measure “thrombotic event and/or death” indicated beneficial effects of pre-existing OAC. We did not evaluate anticoagulation regimes during potential hospital stays. While there may be patients benefitting from full-dose anticoagulation, in general prophylactic or moderate dose might be sufficient. Current studies are ongoing [37].

Our results were independent of the clinical stage and course of COVID-19. Our findings need validation in other COVID-19 cohorts or larger registries that can confirm this hypothesis and should not inform directly on the management of patients with COVID-19. However, they support the observations made by many groups that anticoagulation may be beneficial for most patients with COVID-19.

## Data Availability

Data is available from the authors or the LEOSS study group.
